# Should statin guidelines consider patient preferences? Eliciting preferences of benefit and harm outcomes of statins for primary prevention of cardiovascular disease in the sub-Saharan African and European contexts

**DOI:** 10.1186/s12872-018-0838-9

**Published:** 2018-05-18

**Authors:** Henock G. Yebyo, Hélène E. Aschmann, Tsung Yu, Milo A. Puhan

**Affiliations:** 1 Department of Epidemiology, Biostatistics & Prevention Institute, University of Zurich, Hirschengraben 84, CH-8001 Zurich, Switzerland; 2 School of Public Health, College of Health Sciences, Mekelle University, Ayder, Mekelle, Ethiopia; 3 Department of Public Health, China Medical University, Taichung, Taiwan

**Keywords:** Preferences, Statins, Benefit harm outcomes, Primary prevention, Cardiovascular disease, Clinical guidelines

## Abstract

**Background:**

Patient preferences are key parameters to evaluate benefit-harm balance of statins for primary prevention but they are not readily available to guideline developers and decision makers. Our study aimed to elicit patient preferences for benefit and harm outcomes related to use of statins for primary cardiovascular disease prevention and to examine how the preferences differ across economically and socio–culturally different environments.

**Methods:**

We conducted preference-eliciting surveys using best-worst scaling designed with a balanced incomplete-block design (BIBD) on 13 statins-related outcomes on 220 people in Ethiopia and Switzerland. The participants made tradeoff decisions and selected the most and least worrisome outcomes concurrently from each scenario generated using the BIBD. The design yielded 34,320 implied paired-comparisons and 2860 paired-responses as unit of analysis for eliciting the preferences that were analyzed using a conditional-logit model on a relative scale and surface under the cumulative ranking curve from multivariate random-effects meta-analysis model on a scale of 0 to 1.

**Results:**

There was high internal consistency of responses and minimal amount of measurement error in both surveys. Severe stroke was the most worrisome outcome with a ceiling preference of 1 (on 0 to 1 scale) followed by severe myocardial infarction, 0.913 (95% CI, 0.889–0.943), and cancer, 0.846 (0.829–0.855); while treatment discontinuation, 0.090 (0.023–0.123), and nausea/headache, 0.060 (0.034–0.094) were the least worrisome outcomes. Preferences were similar between Ethiopia and Switzerland with overlapping uncertainty intervals and concordance correlation of 0.97 (0.90–0.99).

**Conclusions:**

Our study provides much needed empirical evidence on preferences that help clinical guidelines consider for weighing the benefit and harm outcomes when recommending for or against statins for primary prevention of cardiovascular disease. The preferences are consistent across the disparate settings; however, we recommend inclusion of more countries in future studies to ensure the generalizability of the preferences to all environments.

**Electronic supplementary material:**

The online version of this article (10.1186/s12872-018-0838-9) contains supplementary material, which is available to authorized users.

## Background

Statins are among the most widely used drugs for primary and secondary prevention of cardiovascular disease (CVD) [1]. Current clinical guidelines have progressively lowered the threshold to initiate statins for primary prevention to 7.5–10% 10-year risk for CVD [2]. As a result, millions of healthy people may be indicated for statins as life-long medication. According to the American College of Cardiology/American Heart Association guideline, for example, 24% of the U.S. population is ought to use statins [3]. Until recently, the use of statins has been limited to affluent regions despite the fact that three-fourths of global CVD deaths occur in low- and middle-income countries [4, 5]. However, the recently improved availability of less expensive generic statins has made them widely accessible to the developing world as well [6].

In spite of their wider use, it is remarkable that existing clinical guidelines do not explicitly assess whether the harms related to statins are trivial enough to be ignored or offset their benefits [7]. Randomized controlled trials (RCTs) have shown statins’ efficacy in reducing risk of new CVD but also reported harmful effects, such as type 2 diabetes, cancer, and hepatic and renal dysfunctions [8, 9]. Clinical guidelines heavily rely on relative effects of statins, which alone cannot justify their use [1, 10]. It is essential to take into account absolute risks of benefit and harm outcomes related to the drugs calculated from the number of prevented outcomes or caused harmful events in people taking statins versus people not taking statins [11]. Moreover, considering social context and people’s preferences is fundamental to move forward to informed medical practices [12]. Some outcomes are more important than others according to the preferences of individuals and thus need to be weighted accordingly when estimating the benefit-harm balance of statins and framing recommendations. Advanced methods that analyze all key parameters–relative effect size, baseline risk and preferences–to quantitatively assess benefit-harm balance have become available that go far beyond the number needed to treat and number needed to harm [11, 13].

While evidence on treatment effects and baseline risks is fairly available, evidence on the preferences, which is a defining parameter to perform benefit-harm analysis, is mostly lacking [14]. Some studies, including the European and Global Burden of Diseases (GBD), aiming at measuring disability-adjusted life-years (DALYs), estimated disability weights–similar to preference weights–for a wide array of diseases [15, 16]. These estimates, however, lack specificity with respect to certain clinical decision contexts, as in the case of prescribing statins for primary prevention of CVD. Furthermore, preferences may vary across populations living in different health systems and social-cultural environments, which may have a substantial impact on the benefit-harm balance of statins and guideline developments [15, 17]. In light of these, we aimed to elicit preferences for benefit and harm outcomes related to the use of statins with emphasis on primary prevention of CVD, and compare preferences between two populations in Sub-Saharan Africa and Europe who live in greatly differing settings in terms of health systems and socio-cultural perspectives.

## Methods

### Study design and participants

We conducted a preference-eliciting survey in Mekelle, Ethiopia, and Zurich, Switzerland from September to December 2016. We considered these environments that differ in terms of health system, socio-cultural and economical statuses, and hence to serve well for comparison of the preferences. Participants had to be 40 years or older and without a history of CVD events. We did not apply any other in- or exclusion criteria.

We obtained the preferences on the predetermined salient benefit and harm outcomes related to statins in the survey using face-to-face interviews. In Ethiopia, the survey was conducted on a home-to-home basis whose respondents were sampled using a computer-assisted random number generator from an existing sampling frame designed for a WHO-stepwise survey. The randomly selected households were visited at convenient times and revisited up to three times when the eligible respondents were not available. The respondents were provided a mobile card for their time that was worth *CHF* 2.50, an amount equivalent to the market value of the compensation provided in Switzerland. In Zurich, we invited participants from people who visited the travel clinic of the University of Zurich. The walk-in clinic renders service of pre-travel advice on preventive measures including vaccinations, to around 20,000 travelers each year. We considered the setting of the travel clinic to provide a good source for recruitment of participants since the demographic profile of visitors to the clinic is similar to that of the Swiss general population [18]. We thus presumed that these individuals represent the general population quite well. The study participants were interviewed face-to-face. They were compensated with a free consultation at the travel clinic that was worth *CHF* 50.

### Questionnaire design and procedure

We designed our study to yield preference weights for a higher-level decision–i.e., to help clinical guideline developers to assess benefit-harm balance of statins, for which knowledge about preference is essential. Since guideline developers have to rely on the published evidence on the effectiveness of statins, we decided to include 13 statin-associated benefit ad harm outcomes in the survey that were reported in RCTs/meta-analyses [8, 9, 19, 20]. The starting point for measuring preferences is a clear definition of the outcomes to be measured because the different outcomes have wide spectrum of manifestation. For example, the statin-associated muscle adverse effects have ranges of presentation from myalgia to rhabdomyolysis [21]. In addition, the participants may not be equally familiar with the medical terms and thus could have different perceptions on the outcomes, which would influence eliciting the preferences [12]. Hence, we used specific lay descriptions for each outcome, instead of the medical terms, which were constructed as a function of health loss–clinical features, treatment modalities, and functional consequences or prognosis–on which the respondents relied to express their preferences (Additional file 1). We tried to simulate a typical manifestation for each outcome while being fully aware that this simplifies reality. We consulted clinical and methods experts to evaluate the lay descriptions. We stated the descriptions using as little technical terms as possible and supplemented anonymous pictures that would help respondents easily select their preferred outcome.

We originally developed the questionnaire in English and then translated it into Tigrigna and German–the local languages in the respective study sites. We consulted bilingual speakers and experts familiar with preference studies to check for consistency and appropriateness of the lay descriptions. We piloted the questionnaires twice in both sites, first on 20 individuals and then on 10 others in the second round of the pilot that helped rephrase the vignettes of the outcomes, and simplify the medical terms. Members of the research and additionally recruited health personnel conducted the interviews. The recruits were trained, overseen, and assisted when the need arose.

The preference questions were designed using the object case Best-Worst Scaling (BWS) method [22], by which participants had to select a pair of best and worst outcomes at a time from presented scenarios. The BWS measures utility or preferences efficiently from fewer respondents. It overcomes the methodological and psychometric weakness of other methods, including visual analogue scale (VAS), pair comparisons, and time tradeoff [23, 24]. In our study, the terms ‘best’ and ‘worst’ refer to the most and least worrisome clinical outcome, respectively. We used the Balanced Incomplete Block Design (BIBD) to get efficiently designed choice sets (Additional file 1). This provided 13 scenarios, with four outcomes in each scenario, to be answered per respondent, where every outcome appeared in four different scenarios and coexisted with another one just once. This design yielded 2860 paired-responses and 34,320 implied-comparisons in the pooled data.

Before they started the BWS procedure, respondents were asked to express their perceived severity using VAS for each of the 13 outcomes in order to familiarize them with the descriptions. This also offered a comparison of the preferences from the VAS with BWS.

Mekelle University, Ethiopia and the University of Zurich granted the study an expedited approval. The study did not involve any physical or laboratory examination, nor did it collect any respondents’ identifiers, and thus we obtained oral consent from each participant after they were briefed about the study’s purpose and procedure, and as well as the confidentiality of the anonymized data.

### Sample size

There is no standard sample size estimation method for BWS studies [25]. We reviewed studies that used BWS and took the median sample size of 220 (this included 10% contingency for non-responses) from studies with similar number of choice sets [22, 26].

### Statistical analysis

We used R (3.2.2) and STATA (13.0) for data analyses and SAS (9.4) to generate the BIBD. First, we used R algorithms to convert the respondents’ dataset into structurally convenient data frame for preference analyses. We assigned 1 for an outcome selected as most worrisome, − 1 for least worrisome, and 0 unless selected otherwise.

We estimated the preferences in log-transformed coefficients (log-odds) and probability terms using different analysis ways; thereafter, we refer to these as relative preferences, and preference weights, respectively. We ran conditional logit-regression models on the 34,320, 18,720, and 15,600 paired-comparisons on the pooled and survey-specific data to get the relative preference values for the outcomes. Unlike standard analyses, this method modeled response as a function of differences in preferences, that indicates utility or preference relations [27] and took into account the correlation of responses within an individual and a scenario. The result from this method explains the relationship with microeconomic theory, which has implications for statistical inference [28]. The model yields preferences on log-scaled linear line on which the different outcomes take relative positions that imply their relative preferences or importance.

We also estimated preferences weights in probability terms; i.e., the probability of an outcome being selected as most worrisome given the rest comparators. First we calculated standardized mean of frequencies of outcome selected as ‘best’ minus ‘worst’ (B–W) scores and standard deviation for each pair of responses. We then ran multiple treatment comparison method (i.e., multivariate random-effects meta-analysis model) on the outcomes to obtain summary of standardized mean differences for the B–W scores for each outcome. With flat priors and posterior normal distribution of the standardized mean difference of B–W scores for each pair of responses and variance equal to the frequentist estimates from the above results, we calculated the probability that an outcome is selected as the most worrisome, the second worrisome, and so on using Markov chain Monte Carlo method in the Bayesian model. This yielded the probability of being most worrisome for each outcome in every trial. We then estimated the surface under the cumulative ranking curve for each outcome in probability terms as shown in Fig. A of the Additional file 1. A preference weight of 1 means that the outcome is certain to be the most worrisome while 0 corresponds to the outcome to be the least worrisome. To estimate confidence intervals, we simulated normal random variates with means defined by the preference weights and variance by the between survey estimates. We then drew 1000 bootstrap samples and repeated the means for each sample. We took the 2.5th and 97.5th percentiles in the distribution about each preference weight.

We assessed measurement error and internal consistency within and between the surveys by portraying a heat map of response probability for the 13 × 13 paired-comparisons. We also ran linear regression to test if survey site and socio-demographic factors affected the preferences.

## Results

### Characteristics of respondents

We obtained responses from all the 220 participants. Table 1 shows socio-demographic and other characteristics of respondents by survey site. Respondents from Switzerland had a higher proportion of educational attainment and employment than those from Ethiopia. While age distribution was truncated by sampling eligibility at 40 years, the participants were older and the probability of morbidity was higher in Switzerland.

**Table 1:** Characteristics of participants involved in the preference eliciting study

Characteristics	Pooled (*n* = 220)	Ethiopia (*n* = 100)	Switzerland (*n* = 120)
Sex			
Male, n(%)	111 (50.4)	54 (54.0)	57 (47.5)
Female	109 (49.6)	46 (46.0)	63 (52.5)
Age			
Mean (SD)	52.9 (0.6)	49.7 (0.7)	55.6 (0.8)
40–64	195 (88.6)	96 (96.0)	99 (82.5)
≥ 65–81	25 (11.4)	4 (4.0)	21 (17.5)
Education			
Mean in years (SD)	10.12 (0.4)	6.55 (0.6)	13.1 (0.4)
None	25 (11.4)	25 (25.0)	0
Primary	46 (20.9)	42 (42.0)	4 (3.3)
Secondary	70 (31.8)	16 (16.0)	54 (45.0)
Higher	79 (35.9)	17 (17.0)	62 (51.7)
Job			
Salaried	107 (48.2)	35 (35.0)	71 (59.2)
Own business	46 (20.9)	24 (24.0)	22 (18.3)
Pensioned	28 (12.7)	4 (4.0)	24 (20.0)
No job	39 (18.2)	37 (37.0)	3 (2.5)
Current or previous statin users	20 (9.1)	8(8.0)	12(10.0)
Co-living person			
Alone	48 (21.8)	11 (11.0)	37 (30.8)
Family	171 (78.2)	89 (89.0)	83 (69.2)
Respondents understand the content and procedure of the questionnaire			
Strongly agree	83 (37.7)	7 (7.0)	76 (63.4)
Agree	116 (52.7)	77 (77.0)	39 (32.5)
Neither	20 (9.1)	16 (16.0)	4 (3.3)
Disagree	1 (0.5)	0	1 (0.8)
Morbidity			
None	149 (67.7)	81 (81.0)	68 (56.7)
Yes ^a^	71 (32.3)	19 (19.0)	52 (43.3)

^a^Hypertension, type 2 diabetes, join/muscle disease, cancer, psychiatric disease were most frequently reported

### Consistency of responses

**Fig. 1:**
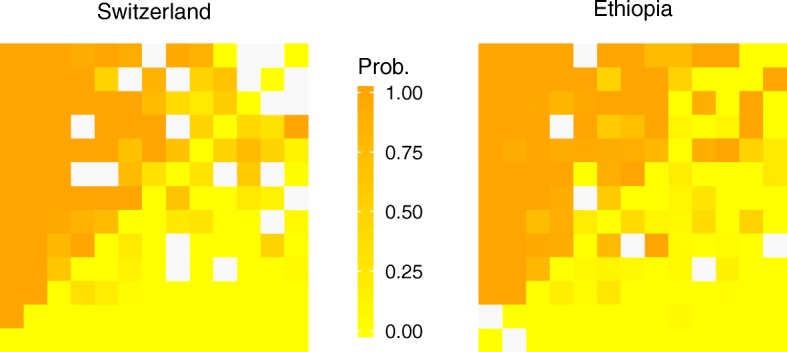
Heat maps indicating consistency of responses. The heat maps show the probability of being selected as most worrisome of each comparison for the 13 × 13 possible combinations of the outcomes. Each cell indicates the probability that the respondents selected the first comparator in a pair as most worrisome. The matrix of the probability is arranged from zero to one, which corresponds to yellow and orange colors, respectively. Except few randomly assorted colors, the visually smooth transition from yellow at the right lower corner to orange at the left upper corner of the maps indicates a small amount of measurement error and high internal consistency. The white patches indicate there were no actual responses corresponding to the pairs; note that this doesn’t mean non-response

Figure 1 shows heat maps of probability for the 13 × 13 possible combinations of outcomes for the survey-specific data. Each cell indicates the probability that respondents selected the first comparator in a pair as the most worrisome. The matrix of the probabilities is arranged from 0 to 1, which corresponds to yellow and orange, respectively. The smooth transition from yellow at the right lower corner to orange at the left upper corner of the map indicated a small amount of measurement error and high internal consistency of the responses within and between the sites. In support to this, we included a question that asked respondents how difficult the descriptions and the BWS procedure were to understand. Eighty four percent (84/100) of the respondents in Ethiopia and 96% (115/120) in Switzerland reported that they well understood the questions and the procedures of the study; where this slight difference corresponded to the slightly less consistency on the heat map from Ethiopia.

### Preferences

The relative preference values (log-odds) of the outcomes compared to the treatment discontinuation along with 95% CI’s are shown in Table 2. Severe stroke, severe myocardial infarction (MI), and cancer were the three most worrisome outcomes. The least worrisome outcomes were treatment discontinuation and nausea/headache. The orders of the relative preferences of all outcomes were consistent across the three models (corresponding to the surveys in Ethiopia, Switzerland and the pooled data) with precise confidence intervals. Since relative scales are not appropriate for comparison, we normalized the values and found that the preferences were similar between Ethiopia and Switzerland. On the relative scale, two point estimates showed reversal in order; i.e., in Ethiopia, heart failure was preferred to moderate MI, and acute kidney failure to unstable angina as compared to the Swiss data. However, the uncertainty intervals ruled out the possibility of flip of orders. The uncertainty intervals of the survey-specific data included the null value for nausea/headache, which suggest respondents had similar preferences for nausea/headache and the treatment discontinuation. In the pooled data, the interval did not contain the null value probably due to the doubled sample size.

**Table 2:** Relative preference values and preference weights of the pooled and separate surveys

	Pooled data		Ethiopia		Switzerland	
Benefit or harm outcomes related to statins	Coefficient (95% CI) Pairs = 34,320	Preference weight (95% CI)	Coefficient (95% CI (Pairs = 15,600)	Preference weight (95% CI)	Coefficient (95% CI) (Pairs = 18,720)	Preference weight (95% CI)
Severe stroke	6.1 (5.8–6.4)	1.00 (0.997–1.00)^b^	5.3 (4.9–5.7)	0.999 (0.996–1.00) ^b^	7.4 (6.9–7.9)	1.00 (0.997–1.00)^b^
Severe MI	4.6 (4.3–4.8)	0.913 (0.889–0.942)	4.0 (3.7–4.3)	0.903 (0.864–0.941)	5.6 (5.2–6.0)	0.869 (0.848–0.921)
Cancer^a^	3.9 (3.7–4.1)	0.846 (0.829–0.855)	3.6 (3.3–3.9)	0.848 (0.821–0.862)	4.4 (4.0–4.7)	0.859 (0.843–0.880)
Moderate stroke	3.1 (2.9–3.3)	0.735 (0.671–0.802)	2.7 (2.4–3.0)	0.686 (0.600–0.783)	3.8 (3.4–4.6)	0.766 (0.644–0.818)
Moderate MI	2.6 (2.4–2.8)	0.664 (0.611–0.715)	2.1 (1.8–2.4)	0.589 (0.517–0.661)	3.2 (2.9–3.5)	0.645 (0.553–0.692)
Heart failure	2.3 (2.1–2.5)	0.591 (0.534–0.647)	2.5 (2.3–2.8)	0.642 (0.593–0.775)	2.2 (1.9–2.5)	0.576 (0.513–0.655)
Type 2 idabetes^a^	1.7 (1.5–1.9)	0.470 (0.452–0.501)	1.9 (1.6–2.2)	0.557 (0.537–0.608)	1.5 (1.3–1.8)	0.378 (0.347–0.424)
Liver injury^a^	1.4 (1.2–1.5)	0.431 (0.345–0.475)	1.3 (1.0–1.5)	0.382 (0.300–0.489)	1.4 (1.2–1.7)	0.461 (0.350–0.531)
Unstable angina	1.0 (0.8–1.2)	0.236 (0.205–0.257)	0.8 (0.6–1.1)	0.255 (0.230–0.302)	1.3 (1.0–1.5)	0.285 (0.247–0.323)
Acute kidney failure^a^	0.9 (0.7–1.1)	0.236 (0.215–0.252)	1.2 (0.9–1.4)	0.283 (0.239–0.296)	0.7 (0.4–0.9)	0.262 (0.242–0.293)
Myopathy^a^	0.6 (0.4–0.8)	0.230 (0.228–0.238)	0.6 (0.4–0.8)	0.256 (0.254–0.258)	0.6 (0.4–0.8)	0.255 (0.253–0.256)
Nausea/headache^a^	0.2 (0.0–0.4)	0.060 (0.034–0.094)	0.2 (−0.1–0.5)	0.093 (0.060–0.139)	0.2 (0.0–0.5)	0.065 (0.035–0.105)
Treatment discontinuation^a,c^	0.0	0.090 (0.023–0.123)	0.00	0.020 (0.00–0.090)	0.0	0.085 (0.002–0.141)

^a^Harms related to statins

^b^One-sided test since 1 is the ceiling values for a probability scale

^c^There could be more harms that are associated with taking statins, including cognitive or sleep disorders problems, which were not reported in our study. Unless stated distinctly by their names, these harms are included in our study with a collective term ‘side effects’ in the BWS procedure for the sake of clarity to the participants, but reported as ‘treatment discontinuation due to side-effects’ here up in the table and throughout the article

Coefficient Log-scaled coefficients from conditional logit model

The preference weights in probability terms are presented in Table 2. In the models run on the pooled and separate surveys, the preference weights were patterned in a similar way as the orders in the relative scale, and with close magnitudes between Ethiopia and Switzerland. In the pooled data, severe stroke outranked all with preference of 1 followed by severe MI, 0.913 (95% CI 0.889–0.942); cancer, 0.846 (0.829–0.855); moderate stroke, 0.735 (0.671–0.802); and moderate MI, 0.664 (0.611–0.715); whereas nausea/headache, 0.060 (0.034–0.094); treatment discontinuation, 0.090 (0.023–0.123); and myopathy, 0.230 (0.228–0.238) were the least worrisome outcomes.

**Fig. 2:**
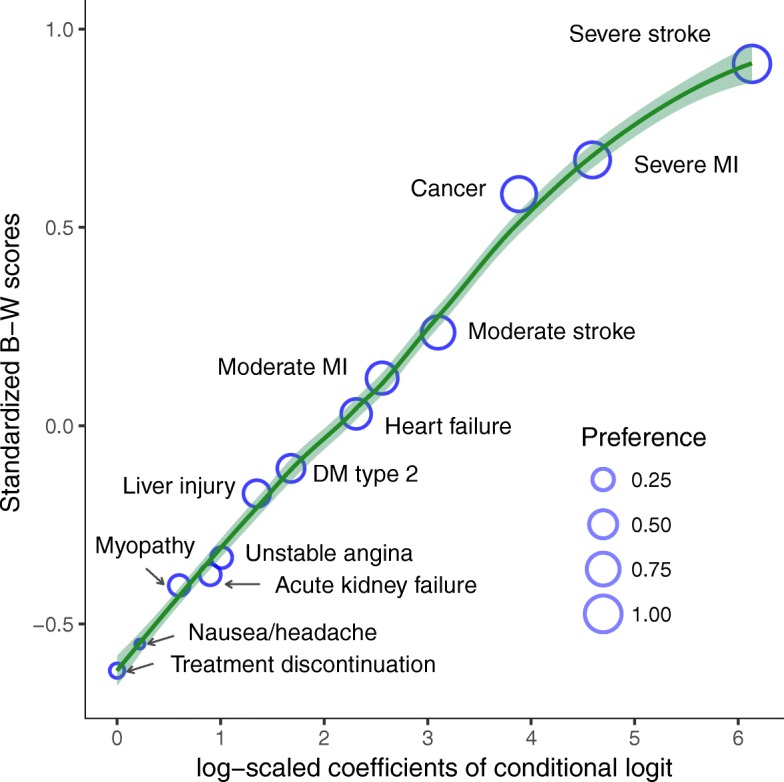
Relationships of preference measurement methods. The smoothed line and increase in circle size portray the relationship between the preference measures

**Fig. 3:**
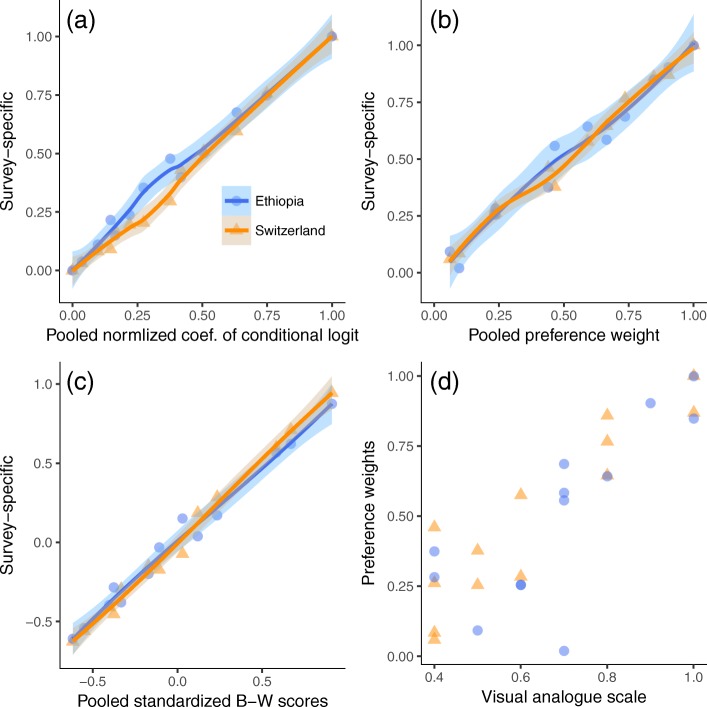
Measurement agreement and consistency of results between the pooled data and specific surveys. Plots (**a**), (**b**) and (**c**) show observed and smoothed uncertainty interval of measurements between surveys. Plot **d** shows correlation between VAS and preference weight. The dots represent the benefit and harm outcomes specified in Fig. 2

We checked the measurement agreement between methods, and the consistency of respective results between the survey sites. Figure 2 shows that the relative preferences, standardized B–W scores and preference weights were linearly related as depicted by the relational line and the corresponding increase in circle areas. Figure 3 presents observed preferences with a local smoothing over survey-specific versus pooled data using different preference scales. The results were consistent between the survey data with overlapping uncertainty intervals of estimates from the linear regression (Additional file 1) and high Lin’s Concordance Correlation Coefficient between the pooled and survey-specific data, 0.98 (95% CI 0.97 to 0.99) for the normalized log-odds and 0.97 (95% CI 0.90–0.99) for both preference weights and standardized B–W scale. Plot ‘d’ presents poor relationship of VAS versus preference weights. The Concordance Correlation Coefficient between these measures was 0.68 (95% CI 0.40–0.85) that indicated VAS was a weak method to quantify preference values.

Reasons most frequently mentioned for influencing respondents’ preference were prognosis, 28% (126 out of 450 responses); severity, 27% (122/450); and curability, 21% (95/450) of the clinical outcomes. The linear regression did not find factors with consistent pattern of influence on the preferences (Additional file 1).

## Discussion

### Summary of results

We found in this study that preferences of respondents from Ethiopia and Switzerland were similar despite large differences in the socio-cultural, health system, and economic contexts. Severe stroke, severe MI and cancers were ranked the worst outcomes while myopathy, nausea/headache, and treatment discontinuation were perceived as the least worrisome outcomes.

### Interpreting and discussing the findings in comparison with the literature

Although the universality of preference weights across culturally, and socio-economically diverse environments is much discussed in the literature [15], our study found consistent preferences for the outcomes between the study sites taken from the Sub-Saharan African and European regions. Indeed, the GBD study that involved wide ranges of clinical outcomes did not show substantially variation of preferences across social contexts [15]. The hypothesis of preference weight variations across settings may stem from previous approaches that had used welfare loss to define the weights instead of health loss [29, 30]. Quantifying preferences with an aim to measure welfare loss is likely to be affected by a society’s level of welfare such as wealth and availability of health insurance. On that account, we predicated that cost or affordability, which greatly varies across the study areas, should not be regarded as a decisive outcome to influence statin prescribing decision because this could bias preferences of people from less affluent regions [31, 32]. Clinical guidelines emphasize on health loss, not welfare loss. Cost of treatment should be managed in a different way once it is established that net benefit of statins outweighs their harms. We controlled potential influences that may contribute to response variation within and across societies by specifying clear constructs of the clinical outcomes in order to ease, standardize, and decrease ambiguity of preference measurements among the participants with varying individual characteristics, such as educational level and awareness. This might have helped attenuate possible inter-personal or cross-environmental preference discrepancies.

We expected a wide range of preference magnitudes between the outcomes and, indeed, we found greatly distinct preference weights in our study ranging from 1 for severe stroke and 0.060 for nausea and headache in the pooled data. The preferences of the other outcomes spread out in between. In the context of prescribing decision, this suggests that severe stroke is the most worrisome and that patients may opt to take statins to prevent risk of severe stroke at the expense of possible unwanted effects. At policy level, these greatly differing values imply that clinical guidelines need to consider the preference values to quantitatively measure the benefit-harm balance of outcomes related to statins. We also presented alternative measures in term of a relative scale to be used in similar way for gradating the importance of the outcomes. For instance, severe stroke was about six times more important than nausea/headache or treatment discontinuation. However, relative scales should not be used to compare the preferences between samples or varying settings, unless they are normalized.

We compared our results with the disability weights published by the European and GBD studies although estimates from these studies are less likely to apply to specific clinical decision-making contexts [15]. The weight for severe stroke, for example, was 0.539 in the GBD study, which significantly differs from the weight we obtained (i.e., 1.00). These studies estimated disability weights for a wide array of diseases and injuries with the aim of measuring DALYs [15]. They compared a given disease with another random disease with high chance of that disease being paired with another more severe one. Consequently, some moderate diseases could get higher weights if randomly paired with a milder, or lower if paired with a severe one, which would to a spurious conclusion. It was not also possible in the GBD study to compare all diseases relative to one another and that the method used could not handle the comparisons in an efficient way [33]. Furthermore, such weights could not be used for all clinical decision-contexts because they could be different if a different decision context is considered, like the statin prescribing scenario, in which preferences are influenced by the number, clinical feature and prognosis of the considered outcomes. For example, there are only about a dozen statin-associated benefit and harm outcomes on which individuals depend to make preference tradeoffs. As a result, the expected preference weights of these outcomes simply cannot be similar with those calculated for other purposes and decision contexts, such as for measuring DALY in the GBD study where many outcomes were considered.

### Implications of findings

The preference weights calculated in our study have important implications for guideline developers and clinicians. No empirical evidence about preferences is available for the statin-associated benefit and harm outcomes and thus clinical guideline developers did not consider when framing the clinical guideline of statins for CVD prevention. There is also a lack of quantitative evaluations of the benefit-harm balance of statins that would needs to include patient-important outcomes, the effects of statins on these outcomes, baseline incidences of the population of interest to calculate absolute effects as well as preference weights for different outcomes [1, 34]. Ignoring preferences and baselines risks could lead to over treatment, especially in primary population where the risk of CVD is low and uncertain. Our study provides preferences values of the outcomes that are much needed for quantitatively assessing the benefit and harm balance of statins. The greatly differing preference values among the outcomes may also give clinicians some guidance for considering patients’ preferences when prescribing statins, and as well as baseline evidence to researchers to develop personalized benefit-harm assessment models.

### Strengths and limitations of the study

Our study has a number of strengths. We clearly defined our decision-context and constructed the descriptions of the clinical outcomes and the answering options in an iterative way to make sure respondents understand them and give us valid preferences. Also, we employed face-to-face interviews to enhance data quality and minimize non-response rate. We pursued efficient methods for comparing all outcomes relative to each other from fewer respondents and analyzed the data with robust methods. Another key strength was testing the hypothesis on possible variation in the preference weights across divergent environments, which is critical in deciding whether setting-specific prescribing practices are needed.

Our study might also have certain limitations regarding the preference estimates. The study sites were not randomly selected. Although we tried to design the tool in a way people would give their preference responses emphasising on the lay descriptions regardless of differences in socio-demographic backgrounds, our data could not rule out the possibility of getting different estimates if we had included other sites. Besides since the descriptions were as short as possible, respondents might give responses based on own perceptions out of the scope of the specified lay descriptions. Generally, there was high internal consistency and low measurement errors, except a few erratic paired-responses from both sites, which may show that a few respondents were skeptical or gave responses based on their perception independent of the lay descriptions.

## Conclusions

Our study provides much needed evidence on preferences related to statins for primary CVD prevention that clinical guideline developers could take into account when developing recommendations. Contrary to popular opinion, our empirical data show that preferences were similar across environments that greatly differ in terms of demographic, socio-cultural and economical perspectives. However, we recommend inclusion of more countries in future studies to make robust conclusion about generalizability of the preferences to more environments.

## Additional file

Additional file 1: This contains an example lay description for chest pain, BIBD design of outcomes, surface under the cumulative ranking curves of benefit and harm outcomes as measure of the preferences, and results of linear regression assessing influence of participants’ characteristics on preference values. (PDF 376 kb)

## Abbreviations

BIBD: Balanced Incomplete Block Design
B–W: Best minus Worst scores
BWS: Best–worst Scaling
CVD: Cardiovascular Disease
DALY: Disability–Adjusted Life Years
GBD: Global Burden of Disease
RCT: Randomized Controlled Trials
VAS: Visual Analogue Scale
