# Supplementary material for: Should statin guidelines consider patient preferences? Eliciting preferences of benefit and harm outcomes of statins for primary prevention of cardiovascular disease in the sub-Saharan African and European contexts
Source: BMC Cardiovasc Disord. 2018 May 18;18:97. doi: 10.1186/s12872-018-0838-9 (PMC5960214; doi:10.1186/s12872-018-0838-9)
Supplement: Supplementary file 1 — This contains an example lay description for chest pain, BIBD design of outcomes, surface under the cumulative ranking curves of benefit and harm outcomes as measure of the preferences, and results of linear regression assessing influence of participants’ characteristics on preference values. (PDF 376 kb) [file 12872_2018_838_MOESM1_ESM.pdf]

Should statin guidelines consider patient preferences? Eliciting preferences of benefits and harms of statins for primary prevention of cardiovascular disease in the Sub-Saharan African and European contexts

Henock G Yebyo[1 2], Hélène E Aschmann[1], Tsung Yu[3], Milo A Puhan[1]

1 Epidemiology, Biostatistics and Prevention Institute, University of Zurich, Zurich, Switzerland

2 School of Public Health, College of Health Sciences, Mekelle University, Mekelle, Ethiopia

3 Department of Public Health, China Medical University, Taiwan

Correspondence to

Prof. Dr. Milo Alan Puhan

Epidemiology, Biostatistics & Prevention Institute

University of Zurich

Hirschengraben 84, CH-8001, Zurich, Switzerland

Email: miloalan.puhan@uzh.ch

Tel. +41 44 634 46 11

Best Worst scaling design

First, we designed a specific construct of the clinical outcomes as functions of clinical features, prognosis, treatment options and recovery status. One example is stated in Box 1. Before the BWS questions, we asked respondents to express their perceived severity using visual analogue scale (VAS) for each of the 13 outcomes in order to familiarize them with the outcomes.

We then provided the participants tradeoff questions that were designed using best worst scaling (BWS) method. The BWS efficiently measures utility or preferences over alternatives from fewer respondents. It overcomes the methodological and psychometric weaknesses of other methods, including likert scale, VAS, pair comparison, person- and time- trade off. This method requires each participant to select preferred outcomes, best and worst, simultaneously in different scenarios. We used the Balanced Incomplete Block Design (BIBD) to get efficiently designed choice sets for the BWS (Table 3). This resulted in four clinical outcomes in separate scenarios from the 13 outcomes (1, 2, 3…13) and 13 total scenarios (questions) (i, ii, iii…xiii) to be answered per participant, where each clinical outcome appeared in four scenarios (colors) and coexisted with another one just once. An example of one scenario appears in Box 2, from which the participants had to select their best and worst outcome.

Box 1 Lay description

Chest pain

You have new chest tightness with squeezing pain that occurs with moderate physical activity, such as walking for more than half a kilometer or walk 10-15 minutes on level ground. After a brief rest, the pain goes away, but it comes again when you have similar activity. Your doctor tells you that you don't have heart attack, but your vessels of your heart are a little tight. You get painkillers and take few other medications and are counseled to adjust your diet and do regular physical activities.

How serious do you think this health problem is?

0 1 2 3 4 5 6 7 8 9 10

Least serious **Most serious**

Table 3. BIBD design

Scenario	Outcome 1	Outcome 2	Outcome 2	Outcome 4
i	3	6	1	12
ii	5	9	7	6
iii	7	8	12	10
iv	11	12	13	9
v	12	5	4	2
vi	1	11	5	8
vii	9	1	10	4
viii	10	13	3	5
ix	13	7	2	1
x	6	4	8	13
xi	2	10	6	11
xii	4	3	11	7
xii	8	2	9	3

Box 2 An example of a scenario of the BWS questions

**Most worrisome** (Choose one)	**Health problems**	**Least worrisome** (Choose one)
☐	Moderate stroke	☐
☐	Heart failure	☐
☐	Moderate heart attack	☐
☐	Cancer	☐

Cumulative line for preference weights

The analysis method for the preference weights is explained in the article. But this figure shows the cumulative ranking line of the weight for each outcome. The exact estimates are included in Table 2 in the main article.

Fig4. Preference weights of outcomes related to use of statins

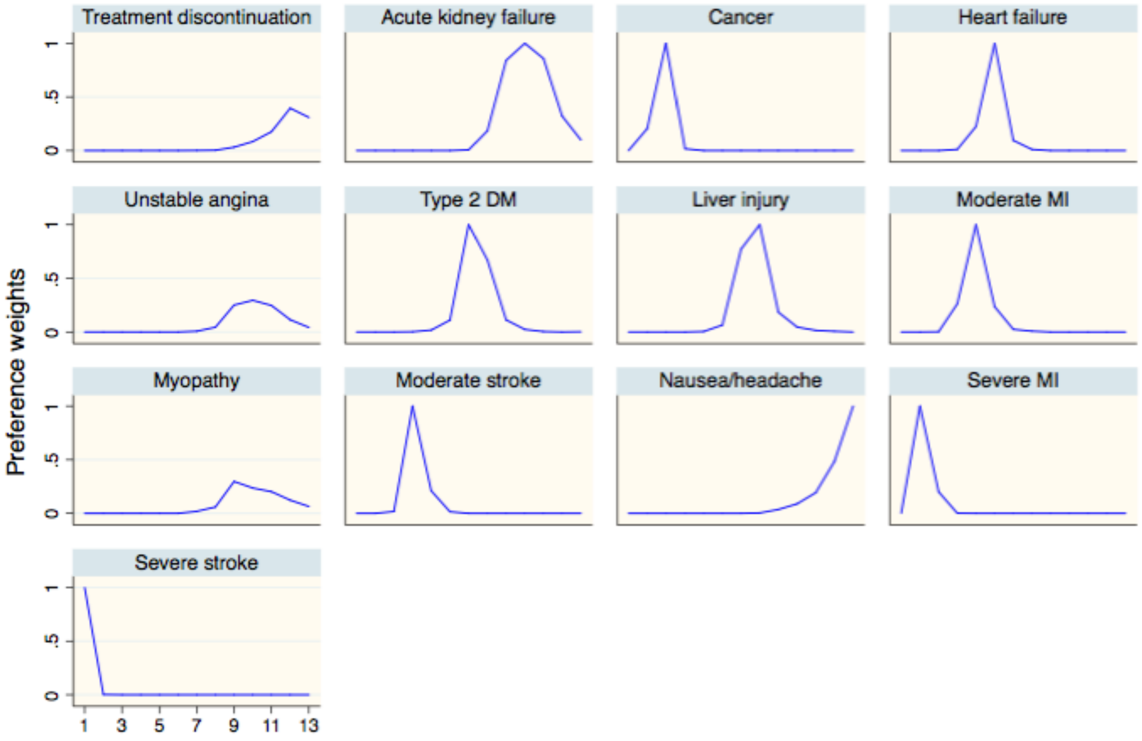

X axes are ranks at which preference weights are estimated

Results of linear regression

Table 4 shows that results from linear regression for all the outcomes. We regressed standardized best minus worst (B-W) scores for each outcome on socio-demographic and other factors using a linear regression to examine associations. But there was no consistent significant association between he factors and the B-W.

Table 4. Association of participants'' characteristics with standardized B-W scores of each clinical outcome

**Characteristics**	**Nausea/Headache**	**Cancer**	**Acute renal Injury**	**DM type 2**	**Severe MI**	**Moderate MI**	**Myopathy**
Site							
Switzerland	0.006 (-0.110, 0.121)	0.017(-0.093, 0.127)	-0.208(-0.325, -0.090)**	-0.136(-0.278, 0.005)	0.083(0.020, 0.146)	0.150(0.073, 0.228)	0.024(-0.104, 0.153)
Ethiopia	Ref						
Sex							
Male	0.059(-0.035, 0.153)	-0.026(-0.116, 0.064)	-0.001(-0.098, 0.095)	-0.087(-0.203, 0.028)	-0.012(-0.063, 0.039)	0.003(-0.060, 0.066)	-0.058(-0.163, 0.047)
Female	Ref						
Job							
Salaried	-0.079(-0.224, 0.065)	0.129(-0.009, 0.267)	0.184(0.036, 0.331)*	0.150(-0.027, 0.327)	0.073(-0.006, 0.152)	0.035(-0.062, 0.132)	-0.078(-0.239, 0.083)
Run own business	-0.099(-0.252, 0.054)	1.74 (-0.017, 0.275)	0.150(-0.007, 0.306)	0.093(-0.095, 0.280)	0.680(-0.055, 0.112)	0.003(-0.100, 0.106)	-0.083(-0.254, 0.088)
Pensioned	-0.084(-0.291, 0.123)	0.265(0.068, 0.462)*	0.262(0.051, 0.473)*	0.319(0.065, 0.572)*	0.008(-0.105, 0.120)	-0.037(-0.176, 0.102)	-0.142(-0.373, 0.089)
No job	Ref						
Comorbidity							
Some	-0.011(-0.109, 0.088)	-1.110(-0.146, 0.041)	0.041(-0.059, 0.141)	0.095(-0.026, 0.572)	-0.130(-0.057, 0.050)	0.039(-0.027, 0.105)	-0.108(-0.217, 0.002)
None	Ref						
Coliving person							
Family member	-0.057(-0.164, 0.050)	0.097(-0.005, 0.199)	-0.014(-0.123, 0.096)	-0.027(-0.158, 0.104)	0.010(-0.048, 0.068)	0.017(-0.054, 0.089)	0.173(0.054, 0.293)**
Alone	Ref						
Age(yrs)	0.001(-0.006, 0.007)	-0.002(-0.011, 0.007)	-0.003(-0.010, 0.003)	-0.010(-0.018, -0.003)*	0.001(-0.002, 0.005)	0.004(0.00, 0.008)	0.002(-0.005, 0.009)
Years of education	-0.002(-0.011, 0.008)	0.542(0.211, 0.973)	-0.003(-0.013, 0.006)	-0.008(-0.020, 0.003)	-0.004(-0.009, 0.002)	-0.006(-0.012, 0.001)	0.007(-0.003, 0.018)

Table 4. Association of participants'' characteristics with standardized B-W scores of each clinical outcome

**Characteristics**	**Liver Injury**	**Heart failure**	**Unstable angina**	**Severe stroke**	**Moderate stroke**	**Treatment discontinuation**
Site						
Switzerland	-0.013(-0.133, 0.106)	-0.199(-0.317, -0.080)**	0.150(0.055, 0.246)	0.050(-0.008, 0.108)	0.133(0.033, 0.233)**	-0.058(-0.184, 0.069)
Ethiopia	Ref					
Sex						
Male	0.035(-0.063, 0.133)	0.088(-0.008, 0.186)	0.029(-0.048, 0.107)	0.011(-0.036, 0.059)	0.006(-0.076, 0.088)	-0.048(-0.151, 0.056)
Female	Ref					
Job						
Salaried	0.121(-0.029, 0.271)	-0.159(-0.308, -0.011)*	-0.065(-0.184, 0.054)	0.021(-0.052, 0.093)	-0.075(-0.201, 0.049)	-0.255(-0.413, -0.096)**
Run own business	-0.013(-0.173, 0.146)	0.002(-0.155, 0.160)	-0.930(-0.186, 0.067)	-0.008(-0.085, 0.069)	-0.037(-0.169, 0.096)	-0.107(-0.275, 0.061)
Pensioned	0.126(-0.089, 0.341)	-0.285(-0.497, -0.072)**	-0.090(-0.260, 0.081)	-0.015(-0.119, 0.089)	-0.195(-0.374, -0.017)	-0.132(-0.359, 0.095)
No job	Ref					
Comorbidity						
Some	0.116(0.014, 0.218)*	-0.073(-0.174, 0.028)	-0.450(-0.1--, 0.062)	-0.005(-0.054, 0.045)	0.046(-0.039, 0.131)	-0.065(-0.173, 0.043)
None	Ref					
Coliving person						
Family member	-0.059(-0.171, 0.052)	-0.082(-0.192, 0.028)	0.035(-0.054, 0.123)	0.021(-0.032, 0.075)	-0.050(-0.143, 0.042)	-0.066(-0.183, 0.052)
Alone	Ref					
Age (yrs)	0.001(-0.006, 0.008)	0.003(-0.003, 0.010)	-0.001(-0.007, 0.004)	0.001(-0.003, 0.004)	0.004(-0.002, 0.009)	0.0004(-0.0007, 0.0007)
Years of education	-0.005(-0.015, 0.005)	0.006(-0.004, 0.015)	-0.003(-0.011, 0.005)	0.003(-0.002, 0.008)	-0.001(-0.009, 0.007)	0.018(0.007, 0.028)**

Ref: Reference group
